# Developing a simple universal hypo-osmotic swelling test (HOST) for assessing sperm membrane integrity in pigs, rabbits, and goats

**DOI:** 10.5455/javar.2025.l913

**Published:** 2025-05-16

**Authors:** Khuong Thi Thanh Tran, Tan Nhat Nguyen, Duy Lam Khanh Nguyen

**Affiliations:** 1 Stem Cell Laboratory, Institute of Food and Biotechnology, Can Tho University, Can Tho City, Vietnam

**Keywords:** Goats, HOST, osmotic concentration, pigs, rabbits, sperm

## Abstract

**Objective:**

The objective of this study was to evaluate the integrity of the plasma membrane for human and other domestic animal spermatozoa by the hypo-osmotic swelling test (HOST).

**Materials and Methods:**

This study was conducted on spermatozoa of three species, including pigs, rabbits, and goats. Three experiments were conducted on three animal species (pigs, rabbits, and goats) to investigate the factors affecting sperm membranes: osmotic concentration (0, 50, 100, and 150 mOsmol) and treatment time (0, 15, 30, 45, 60, and 90 min) at 37°C.

**Results:**

The results showed that treatment time and osmotic concentration influenced the integrity of the sperm membrane in the hypo-osmotic solution. In general, the experiments showed a high rate of sperm reacting to HOST at 50 and 100 mOsmol for 30–45 min. According to this study, the hypo-osmotic solution resulted in a high rate of sperm with swollen tails and a high rate of viable sperm, indicating a correlation between these factors. Therefore, it is necessary to combine both conditions to assess sperm quality. Specifically, the positive rates for the HOST reaction in pig, rabbit, and goat sperm are 46.74%, 58.28%, and 61.95%, respectively.

**Conclusion:**

To sum up, the hypo-osmotic solution with a concentration of 100 mOsmol and an incubation time of 45 min is considered the optimal and most feasible condition that can be used for all three species: pigs, rabbits, and goats.

## Introduction

Sperm quality is critical for the success of artificial insemination (AI) and *in vitro* fertilization (IVF). Traditional parameters for assessing semen quality—sperm morphology, concentration, and motility—while routinely used, have limitations in predictive accuracy and subjectivity [1]. However, because spermatozoa are not examined for membrane integrity, these subjective metrics have been demonstrated to have poor predictive value for tracking testicular function [2]. Advanced techniques such as computer-assisted sperm analysis provide objective assessments but require costly equipment and software, which are often inaccessible to breeding stations and laboratories in resource-limited settings. Consequently, there is an urgent need for alternative methods that are cost-effective, reliable, and simple to perform without sophisticated tools.

The integrity of the sperm plasma membrane is a crucial determinant of sperm viability and fertilization capability. It plays essential roles in various reproductive processes, including sperm activation, the acrosome reaction, and adhesion to the oocyte surface [4,5]. Evaluating membrane integrity offers valuable insights into sperm functionality and fertility potential [6]. The hypo-osmotic swelling test (HOST) is a well-established assay for assessing membrane integrity, as it is simple, cost-effective, and capable of evaluating sperm function by inducing visible morphological changes in biochemically active sperm under hypo-osmotic conditions [7–9].

Although HOST has been applied to humans and several domestic animal species, its use has predominantly been species-specific, with protocols and results tailored to individual species. Each species exhibits unique sperm membrane compositions, leading to variations in their response to hypo-osmotic conditions [10]. However, there is a lack of standardized protocols that can be universally applied across species. Addressing this gap is critical for developing a unified approach that facilitates cross-species applications, particularly for species such as pigs, rabbits, and goats, which are important for agriculture and food security but remain underexplored in the context of HOST optimization.

Current studies on HOST have focused on single-species analysis, leading to a limited understanding of interspecies variations in sperm membrane response to hypo-osmotic conditions. This fragmented approach has hindered the development of a universal protocol for assessing sperm membrane integrity across multiple species. Such a protocol would not only simplify sperm quality assessments but also enable broader applications in reproductive biology, particularly in regions with limited access to advanced reproductive technologies, such as the Mekong Delta.

The primary objective of this study is to develop a universal and cost-effective HOST protocol that can be applied to pigs, rabbits, and goats, identifying optimal conditions of osmotic concentration and incubation time for assessing sperm membrane integrity. This work is novel in its approach to standardizing HOST across multiple species, providing a comparative analysis of species-specific responses and establishing a foundation for cross-species reproductive research. Unlike prior studies, our research contributes to the field by filling the knowledge gap on interspecies HOST optimization and offering practical solutions to improve the efficiency of AI and IVF techniques.

## Materials and Methods

### Ethical approval

Ethical approval was obtained for the animal care, housing, and semen collection procedures, following the guidelines of the Animal Welfare Assessment (CTU-AEC24004 and BQ2022-03/VCNSHTP).

### Chemicals

The chemicals used in the study included citric acid (Sigma, USA), D-glucose (Thermo Fisher Scientific, USA), Eosin Y (Himedia, India), NaHCO₃ (Sigma, USA), NaOH (Sigma, USA), nigrosine (Himedia, India), sodium citrate (Biotech, Vietnam), sucrose (Sigma, USA), and tris-hydroxylmethyl aminomethane (Biobasic, Canada).

### Animals

The male goats and rabbits are raised at the animal experimental farm of the Stem Cell Laboratory, Can Tho University. The animals were carefully cared for and fed 3 times/day according to diet [11,12]. The animals were fully vaccinated against diseases including enterotoxemia, anaplasmosis, lymphadenitis, and foot-and-mouth disease, and their health was monitored regularly. The study performed semen sampling from experimental animals at a frequency of every 3 days for each experimental animal. The imported pig sperm sample was purchased from the pig breeding farm in Hung Loi ward, Ninh Kieu district, Can Tho City.

### Experimental Design

In this study, semen was collected from three male goats and three male rabbits twice a week using an artificial vagina maintained at a temperature of 40°C–42°C. To ensure successful collection, the female was placed in the male’s cage, and the semen collector placed the artificial vagina between the male’s hind legs. In the case of pig semen, it was purchased from the pig breeding farm with semen from 3 male pigs. The collected semen was carefully evaluated for characteristics such as color and pH, as well as semen quality parameters including concentration, motility, and viability.

The semen samples were diluted with TCG medium (250 mM tris-hydroxymethyl aminomethane, 88 mM citric acid, 47 mM D-glucose, and 80 mg/l gentamycin) in a ratio of 1:10, and sperm morphology was examined. The control sample was prepared with semen that had been heat-treated (incubated at 100°C until all sperm were dead). Next, the sperm were placed in HOST solution at osmotic concentrations (0, 50, 100, and 150 mOsmol) in a ratio of 2:8, and the mixture was incubated at 37°C and observed at time points (0, 15, 30, 45, 60, and 90 min). Samples were examined by HOST swelling assessment and sperm viability.

### Assessment of Sperm Concentration

Following the loading of 9 μl of the sample, the counting chamber was given four minutes to acclimate to ambient temperature. A minimum of 200 intact spermatozoa (with entire heads and tails) were counted in each counting chamber under a 40x magnification microscope. Spermatozoa with heads positioned on the dividing line above and to the left of a square were included in the count, whereas those on the line separating two squares were tallied once to prevent double-counting. The WHO criteria [13] were followed to calculate the sperm count.

### Assessment of Sperm Motility

Two wet mounts, each with a depth of about 20 μm, were made on a counting chamber for each sample. Spermatozoa motility was assessed by classifying them into three groups: immotility, non-immotility, and progressive motility. A random counting area was chosen, eliminating areas with only motile spermatozoa present, to guarantee an objective evaluation. In every field, an initial assessment was carried out without waiting for spermatozoa to enter the assessment region. In each wet mount, spermatozoa from a minimum of five fields were counted. Two distinct wet mounts underwent two counts, and the outcomes of the two mounts were compared. The average was determined if the rate of sample variation was within an acceptable range [14].

### Assessment of Sperm Viability

Sperm viability was assessed using the Eosin-Nigrosin method. A volume of 50 µl of the semen sample was mixed with 50 µl of Eosin-Nigrosin solution and allowed to incubate for 30 sec. Subsequently, the mixture was placed on a glass slide and air-dried. Under a microscope, 100 spermatozoa were examined and categorized. Viability spermatozoa were identified by their white appearance or partial red or dark pink staining in the neck region, while the remaining head portion remained unstained. In contrast, dead spermatozoa exhibited a reddish or dark pink coloration in the head region. The rate of viable spermatozoa was calculated based on the observed counts [15].

### Assessment of Sperm Membrane Integrity

The HOST was employed to assess the sample. An Eppendorf tube containing 20 µl of semen sample and 80 µl of HOST at concentrations (0, 50, 100, and 150 mOsmol) (Table 1) solution was placed in a 37°C incubator. After incubation time (0, 15, 30, 45, 60, and 90 min), a 10 µl portion of the mixture was placed on a glass slide for microscopic examination. Spermatozoa with intact membranes exhibited swelling in the tail region, whereas those with compromised membranes did not display any swelling [16].

**Table 1.** Table 1. The mass of sucrose and sodium citrate needed to be mixed at each different concentration of HOST solution, with a volume of 10 ml.

**Osmotic concentration (mOsmol)**	0	50	100	150
**Sodium citrate (gm)**	0	0.0245	0.0490	0.0735
**Sucrose (gm)**	0	0.1712	0.3423	0.5135

### Statistical analysis

R 4.3.1 was used along with Excel to analyze the data. The influence of cysteine concentration was the primary factor investigated. The data were analyzed using a Linear Mixed Model ANOVA, and then mean comparisons between treatments were performed using the Turkey method in the R 4.3.1 software. The mean ± SE of the results is displayed. A high degree of confidence in the acquired data was indicated by the statistical significance, which was established at *p* < 0.05. The R 4.3.1 software was used to create the graphs.

## Results and Discussion

### Fresh Semen Quality

The sperm overall motility, progressive motility, and viability of pigs, rabbits, and goats were shown in Table 2. The results indicated that the average pH was 7.36 for pigs, 6.96 for rabbits, and 6.98 for goats. The semen of pigs, rabbits, and goats had a slightly alkaline pH, in the order of pigs with a pH of 7.2–7.5, rabbits with a pH of 6.5–7.2, and goats with a pH of 6.8–7.5. If the semen has a pH lower or higher than the standard, it is abnormal semen, which is not good for the vitality and fertility of the sperm. The average concentration was 0.51 × 109 for pigs, 0.64 × 109 for rabbits, and 2.65 × 109 for goats. When compared to the WHO [13] standard semen chart, all 3 types of semen have semen concentrations that meet the standard. The average total sperm motility of the 3 species compared to the progressive motility accounted for more than 70% of the total motile sperm. The average sperm viability of pigs was over 75%, and that of rabbits and goats was over 80%, meaning that most of the sperm survived. The initial semen sample evaluation results met the quality conditions to conduct the experiments.

**Table 2.** Table 2. Sperm quality variables (%) recorded in freshly collected pig, rabbit, and goat semen. Data are expressed as mean values ± SEM (*n =* 9).

Animals	Evaluation criteria				
	pH	Concentration (×109 cells/ml)	Overall motility (%)	Progressive motility (%)	Viability (%)
Pigs	7.36 ± 0.05	0.51 ± 2.63	72.24 ± 2.10	65.20 ± 2.81	75.90 ± 0.28
Rabbits	6.96 ± 0.03	0.64 ± 5.94	80.32 ± 0.59	65.74 ± 0.86	88.51 ± 0.68
Goats	6.98 ± 0.02	2.65 ± 2.12	80.87 ± 0.83	66.19 ± 0.93	89.01 ± 1.22

### Effects of incubation time and osmotic concentration on the ability to perform HOST and viability of pig sperm

**Fig. 1.**
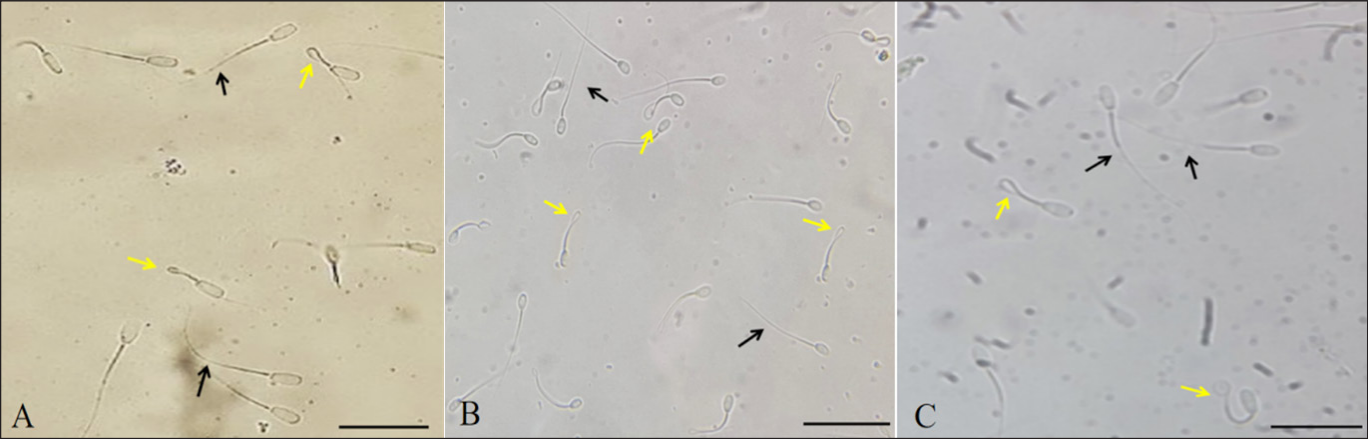
Figure 1. Morphology of sperm in HOST reaction in three species. (A) Pigs, (B) Rabbits, (C) Goats. Each scale bar represents 50 µm, yellow arrows show sperm with HOST reactivity, and black arrows show sperm without HOST reactivity.

The results in Figure 3 showed the swelling response of pig sperm under the influence of incubation time and osmotic pressure. It can be seen that there is an interaction between incubation time and osmotic pressure on the sperm tail bending rate in pigs. The rate of sperm tail bending was highest at 30 min and 0 mOsmol (53.86%) and lowest at 0 min and 0 mOsmol (14.64%); the difference was statistically significant (*p* < 0.05). In general, the concentrations of 0, 50, 100, and 150 mOsmol all showed an increase in the sperm tail bending rate with incubation time up to 60 min, while the concentrations of 50 and 100 mOsmol showed a decrease in the rate of sperm tail bending in the period from 60 to 90 min. Concentrations of 50 and 100 mOsmol showed the highest pig sperm tail bending rate at 45 min and then gradually decreased. The sperm morphology image in the HOST reaction is in Figure 1.

**Fig. 2.**
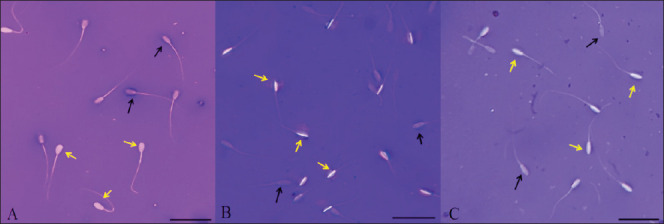
Figure 2. Morphology of sperm in Eosin-Nigrosin staining reaction in three species. (A) Pigs, (B) Rabbits, (C) Goats. Each scale bar represents 50 µm, yellow arrows indicate live sperm, and black arrows indicate dead sperm.

**Fig. 3.**
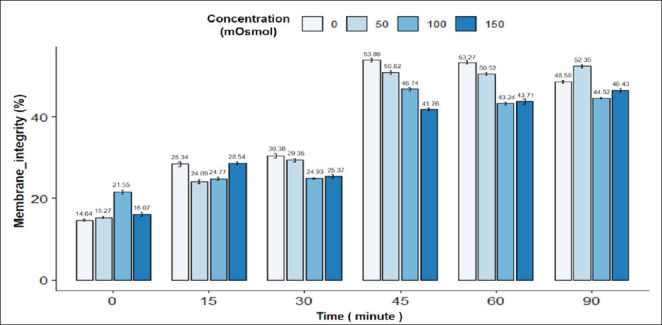
Figure 3. Effect of incubation time and osmotic concentration on HOST (+) reaction of pig semen. Data are expressed as mean values (*n =* 9).

**Fig. 4.**
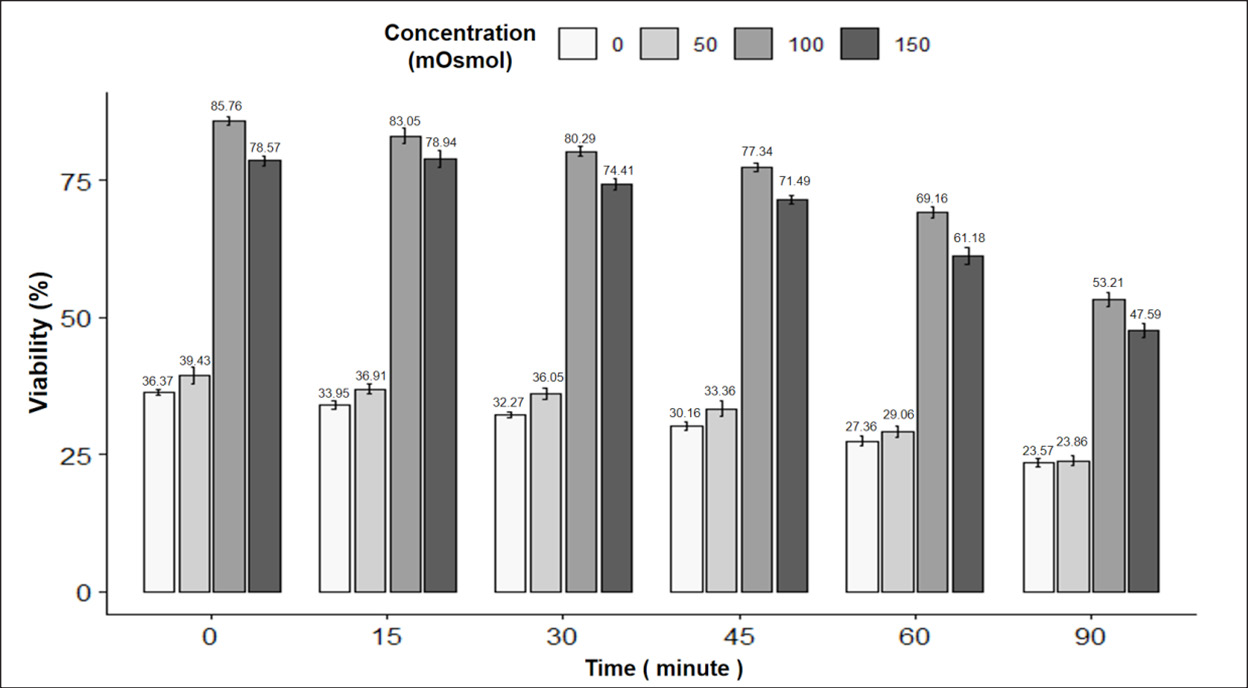
Figure 4. Effect of incubation time and osmotic concentration on viability of pig semen. Data are expressed as mean values (*n =* 9).

Figure 4 shows the results of the viability rate of pig sperm under the influence of incubation time and osmotic pressure. The pig sperm viability rate was highest at the incubation time of 0 min with a concentration of 100 mOsmol (85.76%) and the lowest one (23.57%) at the experiment of 90 min incubation and 0 mOsmol; the difference was statistically significant (*p* < 0.05). It can be seen that at concentrations of 0, 50, 100, and 150 mOsmol, the rate of sperm gradually decreased with incubation time at 37°C. The sperm morphology image in the eosin-nigrosin staining reaction is in Figure 2.

To choose the appropriate osmotic pressure and incubation time for the HOST reaction, the viability and tail swelling ability of the sperm are the deciding factors in choosing which strain has the best sperm tail swelling ability [17]. Therefore, the osmotic pressure and incubation time are selected based on the viability and tail swelling rate of pig sperm. For pig sperm, the rate of tail-curling sperm was highest at the osmolality level of 0 mOsmol and incubation times of 45 and 60 min; however, the sperm viability did not meet WHO standards [13]. Therefore, the incubation time of 45 min with an osmotic concentration of 100 mOsmol was chosen as the optimal experiment to use for pig sperm. Specifically, the viability rate of pig sperm in the 45-min incubation treatment at a concentration of 100 mOSmol was 77.34%.

### Effects of incubation time and osmotic concentration on the ability to perform HOST and viability of rabbit sperm

**Fig. 5.**
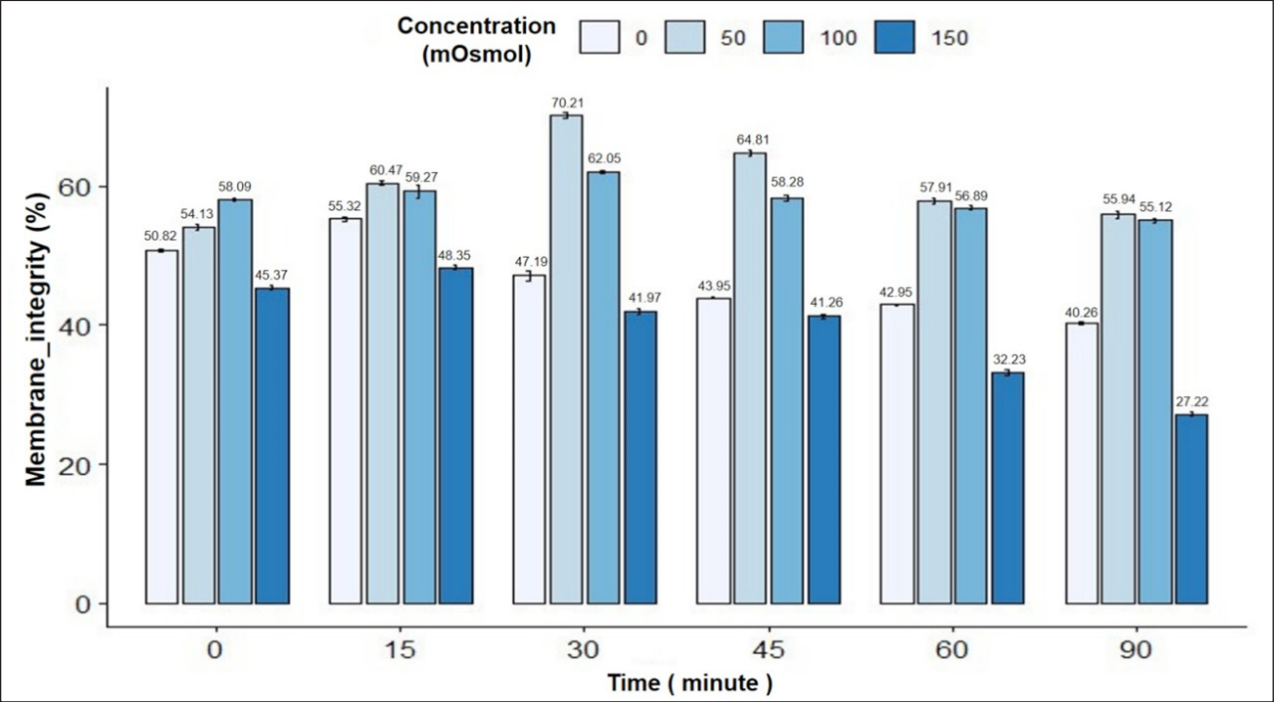
Figure 5. Effect of incubation time and osmotic concentration on HOST (+) reaction of rabbit semen. Data are expressed as mean values (*n =* 9).

The results in Figure 5 showed that in the swelling response of rabbit spermatozoa under the influence of incubation time and osmotic pressure. There was an interaction between incubation time and osmotic pressure on the rate of sperm tail bending in rabbits. The sperm tail bending rate was highest at 30 min and 50 mOsmol (70.21%) and lowest at 150 mOsmol in 90 min of incubation (27.22%); the difference was statistically significant (*p* < 0.05). In general, the concentrations of 0, 50, 100, and 150 mOsmol all showed an increase in the sperm tail bending rate with incubation time up to 30 min and a decrease in the period from 45 to 90 min. Concentrations of 50 and 100 mOsmol showed the highest rate of sperm tail bending at 30 min and then gradually decreased. The sperm morphology image in the HOST reaction is in Figure 1.

**Fig. 6.**
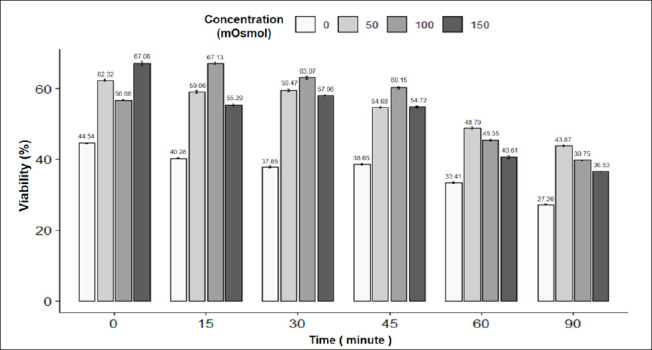
Figure 6. Effect of incubation time and osmotic concentration on the viability of rabbit semen. Data are expressed as mean values (*n =* 9).

The results in Figure 6 showed the results of the viability rate of rabbit spermatozoa under the influence of incubation time and osmotic pressure. The rate of viable sperm was highest at 15 min and 100 mOsmol (67.13%) and lowest at 90 min and 0 mOsmol; the difference was statistically significant (*p* < 0.05). The rate of viable sperm gradually decreased with incubation time, except that the concentration of 50 mOsmol showed an increase in the rate of viable sperm in the time interval from 15 to 30 min, and the concentration of 100 mOsmol increased in the period from 0 to 15 min, both of which then gradually decreased. The sperm morphology image in the eosin-nigrosin staining reaction is in Figure 2.

Combining both evaluation criteria—viability and tail swelling rate of rabbit sperm—the rabbit sperm has the highest tail swelling rate at an osmolality concentration of 50 mOsmol with an incubation time of 30 min. Specifically, the viability rate of pig sperm in the 30-min incubation treatment at a concentration of 50 mOsmol was 59.47% ± 0.61%. Based on the viability of rabbit sperm and tail swelling rate, choose the appropriate osmotic concentration and incubation time. For rabbit sperm, choose the optimal incubation time and osmotic concentration to test the integrity of the rabbit sperm cell membrane at an incubation time of 30 min with an osmotic concentration of 50 mOsmol.

### Effects of incubation time and osmotic concentration on the ability to perform HOST and viability of goat sperm

**Fig. 7.**
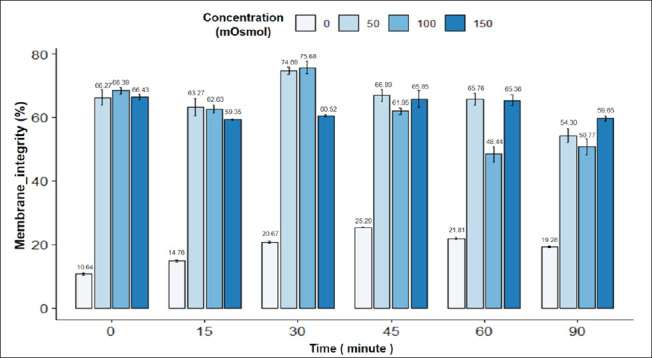
Figure 7. Effect of incubation time and osmotic concentration on HOST (+) reaction of goat semen. Data are expressed as mean values (*n =* 9).

The results in Figure 7 showed that the swelling response of goat sperm was influenced by incubation time and osmotic concentration. There was an interaction between incubation time and osmotic concentration on the tail swelling rate in goat sperm. The highest rate of tail swelling in goat sperm was observed at an incubation time of 30 min with an osmolality of 100 mOsmol (75.68%), while the lowest tail swelling ratio in goat sperm (10.64%) was observed at an incubation time of 0 min with a concentration of 0 mOsmol; the difference was statistically significant (*p* < 0.05). In general, the osmotic concentrations of 0, 50, 100, and 150 mOsmol increased the rate of goat sperm with tail swelling during incubation from 0 min to 45 min, while the osmotic concentrations of 50 and 100 mOsmol showed a gradual decrease in tail swelling ratio in goat sperm throughout 45 to 90 min. Osmotic concentrations of 50 and 100 mOsmol gave the highest rate of sperm with tail swelling at 30 min, then gradually decreased. The sperm morphology image in the HOST reaction is in Figure 1.

**Fig. 8.**
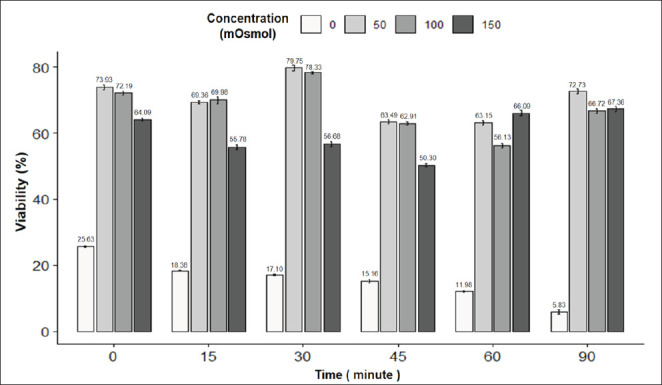
Figure 8. Effect of incubation time and osmotic concentration on viability of goat semen. Data are expressed as mean values (*n =* 9).

Figure 8 presents the results of the viability rate of goat sperm under the influence of incubation time and osmotic concentration. The highest viability rate of sperm was observed at the 30-min time point and 50 mOsmol concentration (79.75%), while the lowest viability rate was observed at the 90-min time point and 0 mOsmol concentration (5.83%); the difference was statistically significant (*p* < 0.05). It can be seen that at the concentrations of 0, 50, 100, and 150 mOsmol, the viability rate of sperm gradually decreased over the incubation time, while the concentrations of 50 and 100 mOsmol showed an increase in the viability rate of sperm over the period from 15 to 30 min. The concentrations of 50 and 100 mOsmol resulted in the highest viability rate of sperm at the 30-min time point, which then gradually decreased. The sperm morphology image in the eosin-nigrosin staining reaction is in Figure 2.

The viability rate and tail swelling rate of sperm are also determining factors in choosing which time and concentration will result in the best sperm tail coiling rate [17]. Therefore, we will combine both indicators to select the appropriate osmotic concentration and incubation time. For goat sperm, the highest sperm tail coiling rate was observed at 30 min and 100 mOsmol concentration. Specifically, the viability rate of pig sperm in the 30-min incubation treatment at a concentration of 100 mOsmol was 78.33% ± 0.76%.

## Discussion

Saccharides and electrolytes are well-known as essential factors in maintaining the functional integrity of sperm membranes, acting as components that regulate osmosis and provide energy for the cell. In this study, we used a hypotonic solution containing sucrose combined with sodium citrate as an electrolyte to create an environment for testing sperm membrane integrity. The mechanism of sucrose in the hypotonic solution is to create an osmotic gradient, leading to the influx of water into the cell and triggering the swelling of the sperm tail. This reaction is particularly more pronounced when using sucrose compared to other sugars, such as fructose, in the study by Fonseca et al. [18].

Notably, the sodium citrate-fructose solution has been shown to be optimal for sperm swelling in both mouse [19] and human [20] sperm, while the sodium citrate-sucrose solution is more suitable for horse [21,22] and rabbit [23] sperm. This reflects the structural differences in sperm membranes between species, particularly the lipid and protein composition of the membrane, which leads to varying responses to different sugars. This study reinforces the view that the choice of sugar should be tailored according to the biological characteristics of each species while also supporting previous reports highlighting the significant impact of membrane properties on sperm response in hypotonic environments [24].

Spermatozoa utilize two main metabolic pathways to generate ATP: oxidative phosphorylation in mitochondria and glycolysis in the cytoplasm [25]. Fructose and glucose are the primary carbon sources for energy production through glycolysis. However, replacing glucose and fructose with sucrose in the hypotonic solution not only provides an alternative energy source but also enhances the osmotic response of the sperm membrane, improving sperm integrity. Excessive reactive oxygen species (ROS) can damage cellular proteins and the lipid structure of the cell membrane, leading to gene mutations and accelerated cell death. In spermatozoa, motility and the integrity of the sperm cell membrane are critical for the acrosome reaction and successful penetration through the inner membrane of the oocyte [4,26]. Mitochondria play a key role in maintaining normal sperm function and energy homeostasis through oxidative phosphorylation and ATP synthase [27]. Under storage conditions, the accumulation of ROS can severely damage the sperm cell membrane and DNA, directly affecting sperm integrity [28]. The addition of antioxidants to the hypotonic solution has been shown to reduce ROS damage, thereby improving sperm viability and quality, as indicated by recent studies [29].

This study also emphasizes the important role of incubation time in testing sperm membrane integrity. The time required to reach osmotic equilibrium in sperm varies among individuals. Some reach equilibrium within one minute, while others take longer, leading to a gradual increase in the HOST (+) rate over time. However, prolonged exposure to the hypotonic environment can cause irreversible changes to intracellular components, membrane structure, and function, resulting in a sharp increase in cell death rates after 45–60 min [30]. Therefore, a 45 min incubation at 37°C was chosen as optimal for all three species in this study.

Regarding osmotic pressure, changes in pressure directly affect the osmotic balance of the cell. Excessively high pressure limits water influx, leading to a low HOST (+) rate, while excessively low pressure induces osmotic stress, resulting in membrane damage. This finding is reported by Holmes et al. [31], who indicated that osmotic pressure higher than the cell’s osmotic pressure does not alter curvilinear velocity. This study identified a solution with an osmolarity of 100 mOsmol as optimal, achieving the highest HOST (+) rate, which aligns with previous studies reporting an optimal osmolarity of 150 mOsmol for Nili Ravi bull sperm [3], 150–200 mOsmol for pig sperm [32], and 125 mOsmol for goat sperm [18]. To select the appropriate osmotic pressure for all three species, it is necessary to combine the evaluation of two factors: the rate of sperm with swollen tails and the rate of viable sperm. Water enters the cell from the outside until equilibrium is reached between the inside and outside of the cell. This study demonstrated the best HOST response at concentrations of 50 and 100 mOsmol, with 100 mOsmol being the recommended concentration for the HOST solution.

The study investigating the effects of incubation time and osmotic pressure on sperm provides valuable insights into the basic assessment of sperm quality, particularly in terms of cell membrane integrity. It confirms the critical role of both osmotic pressure and incubation time in evaluating sperm viability and membrane integrity in a hypotonic solution. However, the study has limitations, particularly the lack of consideration for additional parameters such as acrosome activity and DNA fragmentation, which are essential for a more comprehensive understanding of the effects of hypotonic solutions on sperm quality. Furthermore, to enhance the robustness and applicability of the findings, it is necessary to expand the scope of the research by including a broader range of species, concentrations, and incubation times, thus allowing for a more thorough assessment of sperm quality across different species.

## Conclusion

A hypotonic solution with an osmotic pressure of 100 mOsmol and an incubation time of 45 min is considered the most optimal and suitable for testing sperm cell membrane integrity in the three species of pigs, rabbits, and goats.

## List of abbreviations

AI, artificial insemination

ATP, adenosine triphosphate

HOST, hypo-osmotic swelling

IVF, in vitro fertilization

L, liter

min, minutes

mOsmol, milliosmole

ROS, reactive oxygen species

WHO, World Health Organization

µl, microliter

μm, micrometer.
